# Metagenome-Assembled Genome Sequences of *Acetobacterium* sp. Strain MES1 and *Desulfovibrio* sp. Strain MES5 from a Cathode-Associated Acetogenic Microbial Community

**DOI:** 10.1128/genomeA.00938-17

**Published:** 2017-09-07

**Authors:** Daniel E. Ross, Christopher W. Marshall, Harold D. May, R. Sean Norman

**Affiliations:** a Department of Environmental Health Sciences, University of South Carolina, Columbia, South Carolina, USA; b Department of Microbiology & Immunology, Marine Biomedicine & Environmental Science Center, Medical University of South Carolina, Charleston, South Carolina, USA

## Abstract

Draft genome sequences of *Acetobacterium* sp. strain MES1 and *Desulfovibrio* sp. strain MES5 were obtained from the metagenome of a cathode-associated community enriched within a microbial electrosynthesis system (MES). The draft genome sequences provide insight into the functional potential of these microorganisms within an MES and a foundation for future comparative analyses.

## GENOME ANNOUNCEMENT

Microbial communities inhabiting the cathode of microbial electrosynthesis systems (MES) have been examined for their potential to utilize cathode-derived electrons for CO_2_ conversion into acetate (1, 2). Due to the abundance of *Acetobacterium* spp. (30%) and *Desulfovibrio* spp. (20%) on the cathode surface and their potential importance within the MES, we have determined two draft genome sequences from the cathode metagenome to (i) elucidate the metabolic pathways each genome encodes and (ii) ascertain the role each may play in electrode-dependent CO_2_ fixation.

Metagenome sequencing, assembly, and genome binning were performed as described previously (3, 4). Briefly, a dual-sequencing approach of Illumina MiSeq paired-end sequencing (V2 chemistry, 2 × 250 bp) and Pacific Biosciences sequencing (P4-C2 chemistry) was utilized. MiSeq reads were quality trimmed using the CLC Genomics Workbench and used for PacBio read error correction. The corrected PacBio reads were assembled with Velvet (version 1.2) (5), and *Acetobacterium-*specific contigs were used as the template to map and bin *Acetobacterium*-specific MiSeq reads. Mapped reads were assembled with SPAdes (version 3.5.0) (6), and Velvet-assembled PacBio contigs were merged into the SPAdes assembly as trusted contigs for graph construction, gap closure, and repeat resolution. To obtain the *Desulfovibrio* sp. strain MES5 draft genome sequence, DNA was extracted from anaerobic MES enrichment cultures growing on 1,2-propanediol. Illumina paired-end reads (56% *Desulfovibrio*) were assembled with metaSPAdes (default k-mer values), and contigs were binned with VizBin (7). Both genomes were manually curated for the removal of duplicate single-copy marker genes based upon CheckM (version 1.0.7) output.

The *Acetobacterium* sp. strain MES1 draft genome consists of 61 contigs (*N*_50_, 132,267 bp; longest contig, 440,318 bp; GC%, 44.3%), with a total length of 3,648,609 bp. The 16S rRNA gene was most closely related to *Acetobacterium wieringae* DSM 1911. Genome completeness and contamination were 99.29% and 0.04%, respectively, as assessed by CheckM using the *Eubacteriaceae* marker set (8). Average nucleotide identities between *Acetobacterium* sp. strain MES1 and *A. wieringae*, *Acetobacterium dehalogenans*, *Acetobacterium woodii*, and *Acetobacterium bakii* were 97.44%, 83.58%, 78.95%, and 77.58%, respectively. Similar to other *Acetobacterium* spp., the genome encodes the Wood-Ljungdahl pathway for autotrophic growth, the Rnf complex, electron transfer flavoproteins, and an electron-bifurcating hydrogenase. Pathways for utilization of alternative electron acceptors, such as 1,2-propanediol and 2,3-butanediol, were also present (9, 10).

The *Desulfovibrio* draft genome comprises 3,471,491 bp contained in 52 contigs (*N*_50_, 100,068 bp; GC%, 57.6%), the longest of which is 299,901 bp. The genome was 99.86% complete, with 0.75% contamination (based on 432 *Desulfovibrio*-specific marker genes). A total of 46 contigs (3,229,282 bp) mapped to *D. desulfuricans* subsp. *desulfuricans* ATCC 27774 (E value, 1e^−100^).

Both genomes were annotated with Rapid Annotations using Subsystems Technology (RAST) version 2.0 using the RASTtk pipeline (11, 12) and the NCBI Prokaryotic Genome Annotation Pipeline (http://www.ncbi.nlm.nih.gov/genome/annotation_prok/), and further comparative genomic exploration of each genome is under way.

### Accession number(s).

The *Acetobacterium* sp. strain MES1 and *Desulfovibrio* sp. strain MES5 draft genome sequences have been deposited in the GenBank database under the accession numbers MJUY00000000 and MJUZ00000000 , respectively.
